# A New Standardization of the Bells Test: An Italian Multi-Center Normative Study

**DOI:** 10.3389/fpsyg.2018.02745

**Published:** 2019-01-22

**Authors:** Mauro Mancuso, Alessio Damora, Laura Abbruzzese, Eduardo Navarrete, Benedetta Basagni, Giuseppe Galardi, Marina Caputo, Brunella Bartalini, Michelangelo Bartolo, Chiara Zucchella, Maria C. Carboncini, Simona Dei, Pierluigi Zoccolotti, Gabriella Antonucci, Antonio De Tanti

**Affiliations:** ^1^Tuscany Rehabilitation Clinic, Arezzo, Italy; ^2^National Health Service, Azienda USL Toscana Sud Est, Siena, Italy; ^3^Department of Developmental Psychology and Socialisation, University of Padua, Padua, Italy; ^4^Centro Cardinal Ferrari, Parma, Italy; ^5^Fondazione Istituto G. Giglio di Cefalù, Cefalù, Italy; ^6^Auxilium Vitae Volterra S.p.A., Volterra, Italy; ^7^Department of Rehabilitation, Azienda USL Toscana Nord Ovest, Camaiore, Italy; ^8^Neurorehabilitation Unit, Department of Rehabilitation, Bergamo, Italy; ^9^Neurology Unit, Verona University Hospital, Verona, Italy; ^10^Brain Injury Section, University Hospital Pisana, Pisa, Italy; ^11^Department of Psychology, Sapienza University of Rome, Rome, Italy; ^12^Neuropsychology Centre, Santa Lucia Foundation IRCCS, Rome, Italy

**Keywords:** spatial neglect, bells test, standardization, normative data, diagnosis

## Abstract

**Objective:** The Bells Test is a cancelation task that is widely used for the diagnosis of unilateral spatial neglect (USN). With the aim of fostering more reliable use of this instrument, we set out to develop new norms adjusted for the possible influence of age, gender and education. We worked on the original version of the test.

**Methods:** Normative data were collected from 401 healthy participants aged between 20 and 80 years. Individual factors that could affect performance (i.e., gender, age, and years of education) were considered. We computed several indices on the Bells Test including an asymmetry score, an accuracy score and execution time. Multiple regression analyses (for time measures) and generalized linear models (for accuracy measures) were used to check for the influence of individual predictors of performance on the Bells Test.

**Results:** Data indicated a significant influence of age on the accuracy score and execution time variables and a marginally significant effect of education on the accuracy score variable. Wherever appropriate, cut-offs are provided for the three dependent scores on the Bells Test corrected for age and education.

**Conclusion:** Based on a large normative sample, the present study provides new normative data on the Bells Test, which could lead to its reliable use in the diagnosis of USN.

## Introduction

Unilateral spatial neglect (USN) is commonly defined as the failure to attend or respond to stimuli presented on the side opposite to that of a brain lesion, which cannot be attributed to either sensory or motor defects (Heilman and Watson, 1977). In approximately 40% of patients, neglect becomes chronic and is still present one year after stroke onset (Nijboer et al., 2013). It has functional implications in terms of delayed and difficult rehabilitation gains, higher risk of falls, increasing dependency levels and risk of chronic care in retirement homes (Jehkonen et al., 2000; Paolucci et al., 2001; Buxbaum et al., 2004; Gillen et al., 2005).

Proper diagnosis is important as patients with USN manifest different degrees of spatial impairment, suggesting that it is not an “all-or-none” phenomenon. There is, however, no common criterion for making the diagnosis of USN (Bowen et al., 2013). Thus, more than 60 different tests are used to assess the neglect syndrome (e.g., Menon and Korner-Bitensky, 2004), most of which are paper-and-pencil tasks such as line bisection or visual search/cancelation tasks.

One of these cancelation tasks, i.e., the Bells Test, is widely used for the diagnosis of USN (Gauthier et al., 1989). The patient is required to cross out the bells that are scattered among several different shapes on a sheet of paper. The test is generally easy to administer and score; it allows for a rapid visualization of the location of omissions and for the visuospatial pattern of scanning. Thus, it provides a sensitive estimate of USN when the difference is calculated between the number of targets crossed out on the right side and the number of targets crossed out on the left side (asymmetry score). This index provides information about selective omission of target stimuli in the contralesional hemi-space, a well-known indication of USN. Furthermore, omission of target stimuli on the whole sheet and execution time have proved to be effective in assessing the attention component separately from asymmetrical exploration (Oliveira and Luara, 2016).

In spite of its wide use, the Bells Test has limitations in terms of the scope of its normative data. In fact, as the original norms (Gauthier et al., 1989)refer to a group of only 20 healthy individuals, this could limit the reliability of measurements of USN with this instrument. In a subsequent study, the same research group used a slightly larger sample of 40 healthy individuals; results demonstrated the greater sensitivity of the Bells Test over Albert’s test (Albert, 1973). The absence of distractors in this latter instrument (40 lines drawn in a pseudo-random pattern that the participants had to cross) resulted in a greater sensitivity of the Bells Test to detect the presence of hemineglect, as the presence of distractors induces more omissions errors (Vanier et al., 1990).

It should also be noted that a second version of the test was developed by Vallar et al. (1994). In this study, normative data refer to a larger sample of 212 healthy people and scoring was constituted by omission errors (number of target stimuli omitted), commission errors (number of distractors stimuli wrongly crossed) and time of execution. The performance was influenced by age but not by gender or education. However, this version has different characteristics because the sheet is larger (A3 size) and the stimuli are also enlarged by a factor of about two; thus, data cannot be used as a reference for Gauthier et al. (1989) version, which is the one most used worldwide.

In the present Italian multi-center study, we aimed to collect new normative data on the Bells Test so that it can be used more appropriately in both clinical and research settings. For this purpose, we examined a large sample of healthy individuals in order to be able to evaluate the effects of age, gender and education on performance of the test and to obtain corrected cut-offs for these variables, whenever appropriate, for use with patients with USN.

With an ultimate aim to fortify the interpretation of the Bells Test’s scores, the main objective of this study was to generate new normative data and cut-off values that can be used more appropriately in both clinical and research setting. In a PI/ECO format (Population, Intervention/Exposure, Comparison, Outcome), this study intended to examine the extent to which age, gender and level of education (I/O) affects the performance on the Bells Test (O) in healthy adults (P). We hypothesized that improvement of Bell’s test psychometric properties could allow for a more reliable use of this instrument in the evaluation of USN.

## Materials and Methods

### Sample

Twelve different neuropsychology centers in different parts of Italy participated in the study; they were located in the north (Bergamo, Verona, Parma, and Padua), the center (Pisa, Volterra, and Lucca; two different centers in both Rome and Arezzo) and the south of Italy (Palermo).

We enrolled 412 healthy individuals of both genders (201 M and 211 F), aged between 20 and 80 years. Participants were recruited through local ads and personal contacts. From this original sample, we excluded 11 participants who did not complete the test. Thus, the final sample included 401 healthy individuals stratified into three schooling levels (middle school, high school and college) and 8, 10-year age levels. Sample size was established by applying power analysis for multiple regression (Cohen, 1988) using the pwr package (Champely, 2018) within the R software (R Core Team, 2015) The sample size of 401 individuals satisfies the power analysis with the following parameters: significant level (α) = 0.05; statistical power (1-β) = 0.8; effect size (Cohen’s *f*^2^) = 0.05; number of linear predictors = 3 (for a similar procedure see Brugnolo et al., 2016). The sample composition as a function of age, education (years of schooling) and gender is shown in Table 1.

**Table 1:** Number of participants as a function of age, education (years of schooling) and gender (M-Male and F-Female).

Education	Age											
	18–30		31–40		41–50		51–60		61–70		71–81	
	M	F	M	F	M	F	M	F	M	F	M	F
≤8	6	4	8	10	11	13	13	10	12	11	14	12
9–13	11	12	10	11	18	12	7	8	12	12	15	16
≥14	10	10	17	14	14	12	8	8	10	6	9	15


The following exclusion criteria were adopted:

– signs of previous (or ongoing at the time of the study) neurological and/or psychiatric disorders;
– left-handedness, assessed by the Edinburgh Handedness Inventory (Caplan and Mendoza, 2011);
– signs of cognitive impairment, indicated by a MMSE score lower than 24/30 (Lezak et al., 2004);
– a visual field defect revealed during a clinical examination.

Each participant was assessed with the Bells Test, as well as with other neuropsychological tests, as part of a larger study (in which we also completed the standardization of the Apples Cancellation Test; see Mancuso et al., 2015). Participants did not receive any remuneration for their participation.

The study was approved by the Ethical Committee of the coordination center (Neurological Rehabilitation Unit, USL 9, Grosseto). All participants signed a consent form.

### Tests

The Bells Test (Gauthier et al., 1989; see p. 51 for a copy of the actual stimulus) consists of 315 stimuli randomly distributed on an A4 sized sheet. The stimuli are pseudo-randomly organized in seven different columns: three on the left side, one in the middle and three on the right side. Each column contains 45 stimuli: 40 distractors (common small figures such as houses, horses, etc.) and 5 targets (bells). The paper is placed squarely in front of the participant who is required to identify and cross off the 35 bells scattered among the 280 distractors. The participant is allowed a maximum of 5 min to finish the task.

### Procedure

Each participant was tested in a quiet room with adequate lighting, sitting on a comfortable chair, with both forearms on the table. The sheet of paper was placed exactly in front of the participant and aligned with his mid-sagittal plane. The participant was asked to cross out all the bells on the A4 paper and to ignore the other figures, declaring to the examiner when he has finished. To ensure that the participant understood the task instructions, a practice run-in task was given before the test administration; it included a mixture of oversized targets and distractors displayed on an A4 sheet of paper. The participant was asked to name the elements in order to verify proper object recognition. If the participant finished before all targets were detected, the examiner gave only one encouragement asking: “*Are you sure that all bells are now circled?*” as reported in the original paper by Gauthier et al. (1989). The task was considered finished when the participant stated to have completed the task (in the presence of omissions after the single prompt was given) or at the end of the allotted time. The time taken to complete the task was recorded with a stopwatch.

### Scoring

In accordance with the original version (Gauthier et al., 1989), we divided the scoring sheet into seven columns from left to right; in each one we recorded the total number of circled targets. We also scored omissions of targets, canceled distractors (false alarms) and the difference between the omissions in the three columns on the left and right. The time needed to complete the task was recorded.

The scoring method considers three different scores: an asymmetry score, a total accuracy score and a total time score. The asymmetry score is the difference between the number of targets crossed out on the right side (columns 5–7) and the number of targets crossed out on the left side (columns 1–3). The maximum possible score is +15. Positive values indicate that more targets are crossed-out on the right than on the left side (left-sided neglect) and negative values indicate the opposite (right-sided neglect). The second score is the total number of crossed-out targets and is taken as a measure of selective attention (all target items are considered in this score). The total score ranges from 0 to 35 and indicates how accurate the participant is able to detect targets among distractors.

### Statistical Analyses

In Gauthier et al. (1989) original study, the lowest observed performance in the control group was considered as the cut-off indicating pathological performance, a procedure that could be sensitive to sample variations. The availability of a large sample allowed us to establish cut-offs based on inferential statistical analyses. In the case of accuracy measures (i.e., asymmetry and accuracy scores), distributions were skewed with several individuals showing no error (and no asymmetry). Thus, we chose to analyze the influence of the age and education (measured in terms of years of schooling) predictors using generalized linear models (GLM). In the case of the time measure (execution time), data were analyzed by multiple regression analysis to check for the influence of the predictors age and education. For each of the dependent variables, the confidence interval for distinguishing between a pathological and a normal performance was established based on the cut-off values (with a 95% confidence interval) if the regression was not significant (*p* > 0.05). If the regression was significant, a conversion table was generated to adjust the expected values based on the influence of the significant predictors. Outliers were defined as individual performances above 3 standard deviations from the mean of the group, separately per score. Outliers were omitted from the statistical analyses. Data were analyzed using the R software (R Core Team, 2015).

## Results

Initial analyses indicated the absence of a significant effect of gender in all three dependent variables (*ps* > 0.18); therefore, gender was not considered in subsequent analyses.

There was a small but significant negative correlation between age and education (*r* = -0.17, *p* < 0.001, d.f. = 399), indicating a statistically redundant effect on the analyses.

### Asymmetry Score (Errors)

The data of 6 participants emerged as outliers and were removed from the analyses. The mean asymmetry score was -0.005 (*SD* = 0.92; range = +3 to -3). The mean proportion of responses was similar on both sides (*t* < 1).

The GLM model indicated the absence of any significant effect of *age* (β = 0.001, *z* = 0.06, *p* = 0.94) and *education* (β = 0.11, *z* = 1.13, *p* = 0.25) on the asymmetry score (*ps* > 0.2). Based on a 95% confidence limit (-0.005 + 1.96 × 0.92 = 1.79), a cut-off of 2 was obtained. Therefore, individual performances in which the difference between left and right total omissions was equal to or above 3 should be considered pathological.

### Accuracy Score – Total Omission Errors

Data from 9 participants resulted as outliers and were removed from analyses. The mean accuracy was 1.08 omission errors (*SD* = 1.53; range = 0 – 7). No errors of commission were detected in any of the participants.

The GLM model indicated the presence of a significant effect of *age* (β = 0.025, *z* = 3.85, *p* < 0.001) and a marginally significant effect of *education* (β = -0.042, *z* = -1.92, *p* = 0.054). We chose to include both predictors in the following conversion formula:

expected accuarcy score=−0.83+0.025×age+(−0.042)×education

Then, the maximum accuracy score (above which performance can be considered as pathological) was calculated using the following formula:

maximum accuracy score=expected accuracy score+1.96×1.15⁢ (SD of the residuals)

Table 2 shows expected accuracy scores and maximum accuracy scores as a function of age and education. For convenience of use, the table also reports pathological values; these are calculated based on maximum accuracy scores (after rounding) plus 1; e.g., for individuals in the 20 years of age and 5 years of education slot, the maximum score is 1.71, rounded at 2, which, plus 1, gives a cut-off of pathological performance of 3.

**Table 2:** Expected accuracy and maximum accuracy (expressed as number of errors) according to age and education.

Education	Age												
	20	25	30	35	40	45	50	55	60	65	70	75	80
**Expected accuracy (number of errors)**		
5	–0.54	–0.42	–0.29	–0.17	–0.04	0.09	0.21	0.34	0.46	0.59	0.71	0.84	0.96
8	–0.67	–0.54	–0.42	–0.29	–0.17	–0.04	0.08	0.21	0.33	0.46	0.58	0.71	0.83
13	–0.88	–0.75	–0.63	–0.50	–0.38	–0.25	–0.13	0.00	0.12	0.25	0.37	0.50	0.62
16	–1.0	–0.88	–0.75	–0.63	–0.50	–0.38	–0.25	–0.13	0.0	0.12	0.25	0.37	0.50
**Maximum accuracy (number of errors)**		
5	1.71	1.84	1.96	2.09	2.21	2.34	2.46	2.59	2.71	2.84	2.96	3.09	3.21
8	1.59	1.71	1.84	1.96	2.09	2.21	2.34	2.46	2.59	2.71	2.84	2.96	3.09
13	1.38	1.50	1.63	1.75	1.88	2.00	2.13	2.25	2.38	2.50	2.63	2.75	2.88
16	1.25	1.38	1.50	1.63	1.75	1.88	2.00	2.13	2.25	2.38	2.50	2.63	2.75
**Pathological performance (number of errors)**		
5	3	3	3	3	3	3	3	4	4	4	4	4	4
8	3	3	3	3	3	3	3	3	4	4	4	4	4
13	2	3	3	3	3	3	3	3	3	4	4	4	4
16	2	2	3	3	3	3	3	3	3	3	4	4	4
**Expected execution time (in sec.)**		
	105	108	111	114	116	119	122	125	128	130	133	136	139
**Maximum execution times (in sec.)**		
	200	202	205	208	211	214	216	219	222	225	228	230	233


*Expected execution times (in sec.) and maximum execution times (in sec.) according to age.*

### Execution Time

Data from 3 participants were outliers and were removed from the analyses. The mean execution time score was 122.53 sec. (*SD* = 48.93; range = 11 – 275).

The linear regression model indicated the presence of a significant effect of *age* (β = 0.56, *t* = 3.78, *p* < 0.001) but not of *education* (β = -0.06, *t* = -0.13, *p* = 0.89) on execution time. Based on this outcome, we obtained the following conversion formula:

expected execution time=94.03⁢+0.56×age

Then, the maximum execution time above which performance can be considered as pathological was calculated using the following formula:

maximum execution time=expected execution time+1.96×48.1⁢(SD of the residuals)

Table 2 shows the expected execution times and the maximum execution times as a function of age. Any time above the reported maximum execution times should be considered as pathological.

Statistical data on the effect of age and education on each dependent variable are reported in detail in Table 3.

**Table 3:** Summary of results for the three dependent variables of the Bells Test (asymmetry score, accuracy score and execution time) as a function of age and education.

	Asymmetry score			Accuracy score			Execution time		
	β	*z*	*p*	β	*z*	*p*	β	*t*	*p*
Age	0.001	0.06	0.94	0.025	3.85	0.001	0.56	3.78	0.001
Education	0.11	1.13	0.25	–0.042	–1.92	0.054	–0.06	–0.13	0.89


## Discussion

The results provide solid grounds for identifying pathological performances in the Bells Test. Three predictors (i.e., gender, age and education) and three dependent variables of the Bells Test (i.e., asymmetry score, accuracy score and execution time) were taken into account. As highlighted in previous studies (Gauthier et al., 1989; Vallar et al., 1994; Oliveira and Luara, 2016) the asymmetry score, accuracy score and execution time represent valid indicators of neglect. In particular, the total accuracy score (i.e., the total number of crossed-out targets) is a measure of selective attention indicating how well the participant is able to detect targets among distractors. Furthermore, the asymmetry score (i.e., the difference between omissions in the left and right columns) allows for a more detailed quantification of the difference in target detection. Note that, in the presence of an entirely symmetrical performance in healthy individuals, the cut-off for the asymmetry score can be used both to detect the (more frequent) deficit in the left hemi-space exploration in right brain-damaged patients as well as one in the right hemi-space in left brain-damaged patients (see for instance, Kleinman et al., 2007).

The results indicate a significant effect of the predictor age on the accuracy score and on the execution time variables and a marginally significant effect of the predictor education on the accuracy score. We provide normal performance cut-off points for each of the three dependent variables, controlling for age and education when appropriate.

Our study overcomes previous attempts to provide norms for the Bells Test for several reasons (see Gauthier et al., 1989; Vanier et al., 1990). We examined a larger sample (i.e., 401 healthy participants) and used a more appropriate statistical approach (i.e., GLM or linear regression models depending on the nature of the variables).

However, our research has also some limitations. Specifically, our data were obtained only from healthy Italian individuals and our sample was restricted to right-handed people. As the Bells Test has no verbal components, there is no strong reason to believe that country of origin is an important parameter. However, it would certainly be useful to have corroborating data from healthy individuals of other nationalities to support the generalization of the present norms of the Bells Test. Furthermore, similar to other normative studies (Gauthier et al., 1989; Vanier et al., 1990), we limited our sample to right-handed people only, because left-handers can show atypical patterns of lateralization (Nicholls et al., 2010). However, Willems et al. (2014) emphasized the need to recognize the potential of studying this often-discarded group of research participants. Thus, testing a sample of left-handers would allow examining visuospatial functions in atypically lateralized individuals (Willems et al., 2014).

In sum, these findings allowed us to develop sensitive norms which take into account the effect of age and education wherever appropriate. In the international literature, the Bells Test is one of the most renowned and frequently used tests to evaluate visual hemineglect, since it is simple to administer and allows clinicians quickly detecting the presence of asymmetries in visual search. We propose that using these cut-offs should allow for a more reliable use of the Bells Test in the evaluation of USN.

At the same time, it is important to underscore that neglect is a graded phenomenon and using a single instrument is inevitably prone to the occurrence of false negative (Azouvi et al., 2002). Therefore, it is always advised to use several instruments using different format, such as line bisection, reading, writing, constructive praxis and other cancelation tests, as well as ecologically valid tasks such as the Behavioral subtests of the Behavioral Inattention Scale (BIT; Wilson et al., 1987). This would allow detecting all USN clinical manifestations and minimizing changes of false negatives (Oliveira and Luara, 2016).

## Conclusion

New normative data are reported for the Bells Test. We hope this new standardization will allow researchers and clinicians to make a better use of this widely used tool for the assessment of USN.

## Ethics Statement

This study was carried out in accordance with the recommendations of Ethical Commette of NHS-USL9-GR-Italy, with written informed consent from all subjects. All subjects gave written informed consent in accordance with the Declaration of Helsinki. The protocol was approved by the Ethical Local Committee-NHS USL n.9 Grosseto-Italy.

## Author Contributions

MM, PZ, ADT, and GA designed the experiments. AD, BeB, GG, MC, BrB, MB, CZ, MCC, and SD collected and analyzed the data. MM, PZ, LA, and EN wrote the manuscript. MM, PZ, ADT, and LA provided critical feedback and gave final approval of the version to be published.

## Conflict of Interest Statement

The authors declare that the research was conducted in the absence of any commercial or financial relationships that could be construed as a potential conflict of interest.
